# Interpretable learning algorithms enable pathogenic potential assessment and virulence-associated gene discovery of *Vibrio parahaemolyticus*

**DOI:** 10.3389/fmicb.2026.1832130

**Published:** 2026-05-20

**Authors:** Zhuosheng Liu, Zhuoheng Li, Jiawei Zhang, C. Titus Brown, Luxin Wang

**Affiliations:** 1 Department of Food Science and Technology, University of California, Davis, Davis, CA, United States; 2 Department of Computer Science, University of California, Davis, Davis, CA, United States; 3 Department of Population Health and Reproduction, University of California, Davis, Davis, CA, United States

**Keywords:** deep learning, isolation sources, machine learning, pathogenicity, *Vibrio parahaemolyticus* food safety

## Abstract

The presence of *Vibrio parahaemolyticus* (*Vp*) at various stages of seafood production has adversely affected public health and threatened the sustainability of the industry. Driven by the advancement of next-generation-sequencing technologies and public health data sharing initiative, an increasing volume of public *Vp* genomic data with metadata has become available, which serve as the foundation for building learning models to accurately differentiate isolation sources and further uncover gene-level determinants of pathogenic potential. The primary goal of this study was to develop and validate machine learning (ML) and deep learning (DL) algorithms to differentiate *Vp* strains from different isolation sources (clinical vs. environmental isolates) using pangenome assemblies and achieved robust and precise pathogenic potential prediction of *Vp*. The secondary goal of this study was to obtain critical biological insights revealing pathogenic potential-associated genes contributing to the isolation source difference from these established learning models. Based on the results, the developed learning models demonstrated strong performance, achieving an AUC greater than 0.95 in distinguishing clinical and environmental isolates using pangenome signals. Besides, the gene feature weight analysis from RF revealed the importance of specific accessory genes during *Vp* evolution including but not limited to functional unknown cloud genes, *vspR*, sctC5, and *tdh1*, which provides biological insights as potential future research directions. These findings essentially highlight critical importance of accessory and cloud genes in differentiating clinical and environmental isolates, and provide new insights into how recently acquired genes may contribute to pathogenic evolution of *Vp*. Additionally, the framework demonstrated in this study provides a cost-effective intelligent strategy by leveraging large public genomic datasets to support surveillance and risk assessment of seafood-associated pathogens.

## Introduction

*Vibrio parahaemolyticus* (*Vp*) is a Gram-negative, rod-shaped and halophilic pathogenic bacterium. The wide presence of *Vp* in different production stages of seafood has generated negative impacts on both public health and the seafood industry worldwide, leading to 34,664 illnesses annually in the United States, which is much higher compared with annual illness caused by *Vibrio vulnificus* and other *Vibrio* spp. (96 and 17,564 respectively) based on the 2025 USDA report (Hoffmann et al., 2015). The Economic Research Service of the United States Department of Agriculture (USDA ERS) estimated that the total cost of illnesses caused by *Vp* was increased from $40.7 million in 2013 to $40.7 million in 2018 and *Vp* was ranked as ninth most common illness-cause pathogen out of the 15 listed (Hoffmann and Ahn, 2021). This underscores the need to control *Vp* at different food production stages.

The pathogenic potential of *Vp* is multifactorial, encompassing various virulence factors such as the thermostable direct hemolysin (TDH), TDH-related hemolysin (TRH), and two type III secretion systems (T3SS1 and T3SS2) (Ceccarelli et al., 2013; Liu et al., 2024). While these well-known pathogenic markers are being used as screening tools for identifying pathogenic *Vp*, literature has indicated that *Vp* has a high intraspecific genetic diversity, even for the same serotypes (Alam et al., 2009; Alam et al., 2003; Chen et al., 2017). The availability of next generation-sequencing (NGS) can revolutionize the diagnostics and characterization of infectious diseases and pathogens by overcoming limitations associated with conventional culture-based methods as well as molecular techniques such as polymerase chain reaction (Hilt and Ferrieri, 2022; Jiang et al., 2022; Lecuit and Eloit, 2014). Whole genome sequencing can also reveal emerging virulence factors and elucidate strain-level virulence difference (Ronholm et al., 2016). Currently, there are 2069 completed *Vp* whole genome sequencing data available in the National Center for Biotechnology Information (NCBI) public databases. However, there are several challenges associated with the utilization and interpretation of these data. First, given the volume, complexity and clinical uncertainty of data generated in whole-genome sequencing, expertise is needed for the interpretation of identified genetic variation. Secondly, 10% clinical isolates lacking virulence markers such as *tdh* and *trh*, which are commonly regarded as the major virulence determinants by researchers can still cause disease, which suggests that additional virulence factors may contribute to pathogenicity of *Vp* (Raghunath, 2015; Ronholm et al., 2016). These evidence indicates that relying solely on a limited set of established known virulence genes may underestimate underlying pathogenic potential. Thus, computational approaches leveraging whole genome-wide signals may facilitate identification of additional genetic features contributing to *Vp* pathogenicity.

In recent years, leveraging whole genome sequencing (WGS) data with machine learning (ML) and deep learning (DL) methods have garnered considerable attention and interest (Libbrecht and Noble, 2015) and show potential to address the challenges associated with the use of pangenome data mentioned above. ML approaches, characterized by their ability to identify and learn from patterns and make predictions from data, have emerged as powerful tools to analyze and interpret WGS data (Libbrecht and Noble, 2015). Learning algorithms can identify complex relationships and patterns within the genome, enabling the discovery of genetic variations, functional elements, and associations with virulence serotypes (Im et al., 2021). As a special family of ML algorithms, DL can automatically learn useful features from input data without human involvement, which has been shown to have great performance in tasks such as image recognition and natural language processing (Greener et al., 2022). It is promising to bring these powerful DL tools onto the field of microbial food safety. The integration of ML and DL methods with WGS data has the potential to help researchers better utilize the sequencing data available in databases, enhance our understanding of the pathogenicity basis of bacterial pathogens, facilitate later stage decision making process, and contribute to advancements in final pathogen control.

The goal of this study is to explore the use of ML and DL algorithms in microbial food safety. Particularly, this study aims to leverage ML/DL methods and WGS data to advance the characterization of *Vp* isolated from different sources and identify novel potential pathogenicity features that contribute to clinical diseases, thus further benefiting risk management of *Vp* in the food production chain by ML-driven pathogenic potential surveillance systems.

## Materials and methods

### Pangenome dataset preparation

The overall workflow was visualized in Supplementary Figure 1. Complete genome assemblies of *Vp* were downloaded from NCBI GenBank (^1^total accessed number: 2069). For consistency of genome annotation, the downloaded complete genome assemblies were re-annotated using Prokka (Seemann, 2014). Genome labeling was web-scripted based on genome assembly metadata from NCBI. Isolation source, host information, and sample description information were used for the classification of clinical and environmental isolates in the genome file. *Vp* genomes were qualified for downstream pangenome construction only if their isolation source could be unambiguously ascertained from associated metadata (i.e., isolation source, host information) or corroborated by relevant primary literature linked to the genome records. A total of 1981 isolates were used in ML tasks, of which 872 were clinical isolates and 1,109 were environmental isolates: clinical isolates were used as a proxy for strains associated with human infection with high pathogenic potential (label 1 as high pathogenic potential and 0 as low pathogenic potential). A pangenome was later constructed based on reannotated genome assemblies using Roary (Page et al., 2015). The pangenome constructed by Roary has 101,422 unique genes based on 1980 *Vp* strains.

### Unsupervised clustering algorithm implementation

Unsupervised clustering was first applied to check if the pangenome exhibits distinct grouping patterns that can be recognized by learning algorithms. Four distinct combinations were considered: core genome (sharing among 99% of strains), soft-core genome (sharing among 95% of strains), core genome (core with soft-core) plus shell genome (sharing among 15% of strains), and full pan-genome (including core, soft-core, shell and cloud (sharing among only 0 to 15% of strains)). Initially, Principal Component Analysis (PCA) was employed to visualize the distribution of data points within these matrices. This step aided in understanding the inherent structure and variance present in the datasets. For clustering, we implemented Gaussian Mixture Models (GMM) (Huang, 1998; Reynolds, 2009). The GMM approach was selected for its ability to model data as a mixture of several Gaussian distributions, allowing for the accommodation of clusters of different shapes and sizes. In parallel, the K-MODES algorithm was utilized, because of its suitability for clustering boolean matrices (binary data, represented as 1 or 0). These two methods provided a view of the clustering patterns within the pan-genome data relying only on gene presence/absence without supervised guidance.

### Machine learning model training

This study explored the use of machine learning algorithms. As a single-parametric classification method, K-nearest neighbors (KNN) operates on a simple yet effective principle: the classification of any given data point is predominantly influenced by the majority class among its ‘k’ closest neighbors in the dataset (Guo et al., 2003). The hyperparameter k was set to 5 after testing various values of k, ensuring a balanced blend of precision and generalization. The Support Vector Machine (SVM) with Radial Basis Function was selected for its versatility as well as its proven track record in handling diverse datasets (Boser et al., 1992). Two key parameters were adjusted to optimize the SVM’s performance: the regularization parameter, C, was calibrated to 0.88, striking a balance between achieving accurate classification of training examples and maximizing the decision function’s margin; and the gamma parameter was set at a value of 0.005, limiting the extent to which a single training example could influence the classification. Ensemble tree-based models including Random Forest (RF) and Gradient Boosting Trees (GBT) (100 decision trees, maximum tree depth = None) were fitted to pangenome features (Breiman, 2001; Chen and Guestrin, 2016). These two tree-based ensembles learning methods were chosen for their inherent robustness and ability to navigate multifaceted datasets. Naïve Bayes (NB), a probabilistic classification technique grounded in Bayes’ theorem, was also included in this study. This algorithm is particularly well suited for its simplicity and efficiency in handling high-dimensional data, especially under the assumption of feature independence (Rish, 2001).

### Deep learning model training

For the DL models, two distinct architectures were employed to predict the pathogenic potential of *Vp*. A four fully connected layers Multi-Layer Perceptron (MLP) model was used as baseline. The first layer takes an input of a specified size and outputs to 1,400 nodes. Subsequent layers reduce the dimensionality step-wise: the second layer maps 1,400 nodes to 512, the third maps from 512 to 128, and the final layer maps 128 nodes to the output. Between each fully connected layer, dropout regularization was applied to prevent overfitting. The model uses the ReLU activation function for intermediate layers, promoting non-linearity in the learning process.

The Convolutional Neural Network (CNN) begins with a standard convolutional layer followed by batch normalization and ReLU activation, and a max-pooling layer. The convolutional layers in the model utilize 3×3 filters. It then sequentially connects two residual blocks, increasing the depth and complexity of the model. Residual Block consists of two convolutional layers each followed by batch normalization (He et al., 2016). Post-convolutional layers, the model includes a series of fully connected layers with dropout for regularization. The network uses ReLU activation functions for these layers. The final fully connected layer maps to the number of classes, and binary cross entropy is applied for binary classification. Models were trained for 100 epochs with a batch size of 256. These configurations aimed to strike a balance between model complexity and computational efficiency for accurate pathogenic potential assessment. DL models were trained on a single NVIDIA A100 GPU provided by the UC Davis Data Lab.

### Interpretations of gene feature importance

To identify important gene features contributing to the isolation source differentiation of *Vp*, we used two complementary approaches: feature importance analysis with RF and gradient weighted class activation mapping (Grad-CAM) from a CNN. The RF model ranked gene features based on their contribution to the model’s predictions and highlighting key features critical for distinguishing clinical and environmental isolates. Grad-CAM was employed to visualize CNN predictions by generating heatmaps that pinpoint regions of the input matrix most influential in the model’s decision-making (Selvaraju et al., 2017). Together, these methods provided robust evidence for the roles of specific genes in the classification of *Vp*, combining statistical rigor with model interpretability to support the identification of key gene targets for further investigation.

### Model evaluation and statistical insights

To evaluate the performance of ML and DL prediction models in differentiating *Vp* isolation sources, the following metrics were selected. Accuracy was used to measure the overall correctness of the model, which was calculated as the ratio of correct predictions to the total number of samples. Precision measured the model’s ability with respect to positive predictions, calculated as the ratio of true positive predictions to the total positive predictions made. Recall measured the model’s ability with respect to ground truth positives, which was calculated as the ratio of true positives to the sum of true positives and false negatives. The F1-Score, the harmonic mean of precision and recall, was used as a balancing metric. Additionally, to provide a comprehensive view on model performance on slightly unbalanced dataset, the Area Under the Curve (AUC) of the Receiver Operating Characteristic curve was applied as a performance metric for classification models. Unsupervised clustering was performed using 10 different randomization whereas supervised learning model performance was assessed using 10-fold cross-validation. In each fold, 90% of data as used as train set and 10% of data was used as test set for machine learning models, whereas 80% of data was used train set, 10% of data was used as validation set, and 10% of data was used as test set for deep learning models. All evaluation metrics (accuracy, precision, recall, F1 and AUC score) were reported as the mean ± standard deviation in three decimal places across folds to provide statistical insight into model stability and generalization.

## Results

### Overview of *Vp* pangenome

**Figure 1:**
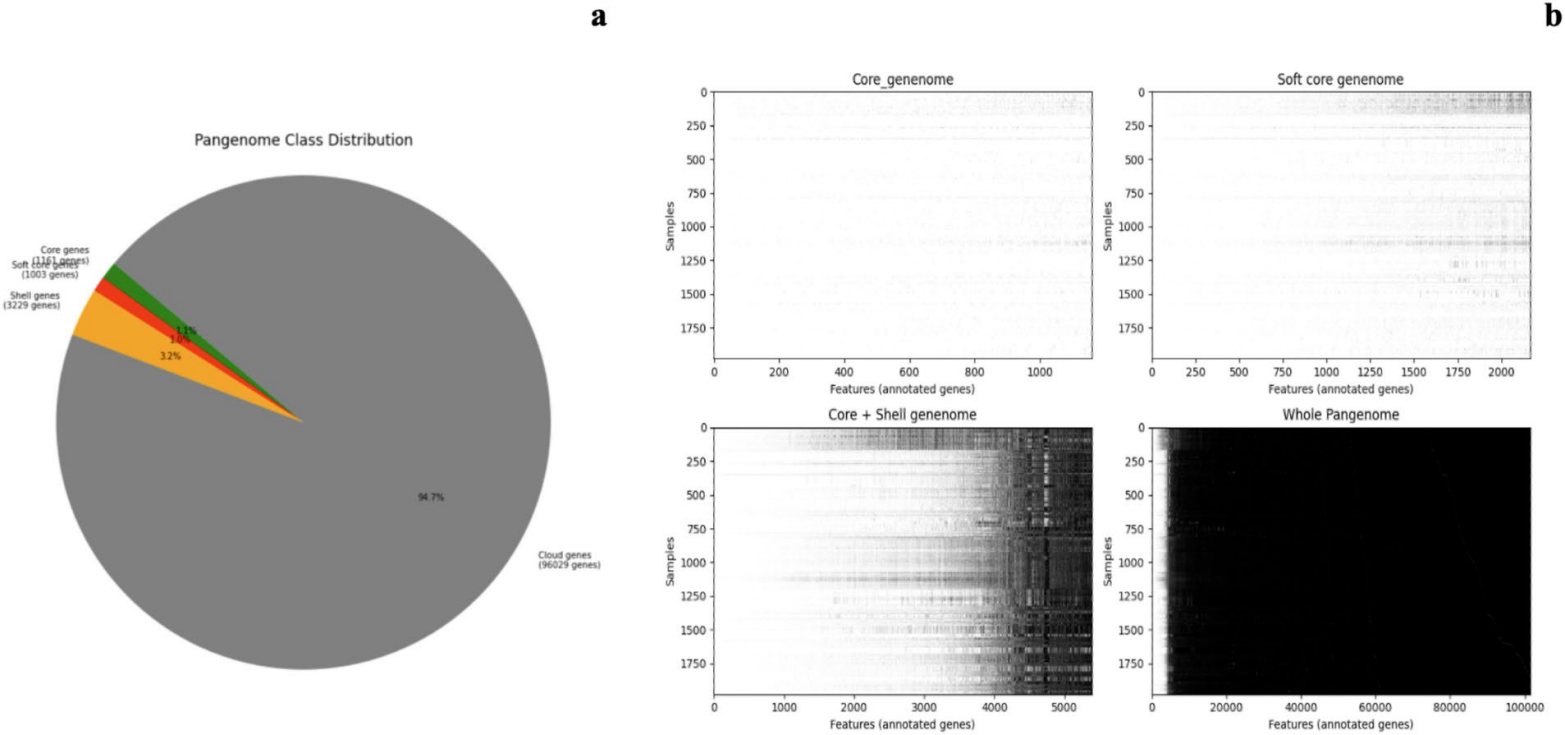
Overview of constructed pangenome of *Vp* based on complete assemblies of 1980 strains. **(a)** The pangenome class distribution shows the proportion of core, soft core, shell, and cloud genes. Core genes (1,161 genes) are present in all strains, indicating their essential role in *Vp* biology. Soft core genes (1,003 genes) are found in 95–99% of strains, suggesting strain-specific adaptations. Shell genes (3,229 genes) occur in 15–95% of strains, likely linked to environmental adaptation and niche colonization. Cloud genes (96,029 genes) appear in fewer than 15% of strains, potentially contributing to unique traits, pathogenicity, or resistance mechanisms. **(b)** Heatmap representations of gene presence-absence across strains in various genome components: core genome, soft core genome, core + shell genome, and whole pangenome. Each row represents a strain, and each column represents a feature (annotated gene), with darker shading indicating gene presence. The whole pangenome reveals the high genetic diversity in *Vp*.

The pangenome was constructed based on the complete genomes of 1,981 *Vp* strains and was classified into four pangenome categories, core, soft-core, shell and cloud, consistent with findings reported by (Sonnenberg et al., 2020b). The distribution and quantity of each categorical pangenome calculated by Roary were captured in Figure 1a and Table 1. The pangenome construction analysis of *Vp* revealed 1,161 core genes present in all strains, serving as the foundation of the genetic identity of *Vp*. Additionally, 1,003 soft-core genes were identified in 95 to 99% of strains. The analysis also uncovered 3,229 shell genes present in 15 to 95% of strains, likely associated with environmental adaptations and niche colonization. The vast genetic diversity of *Vp* is highlighted by 96,029 cloud genes found in fewer than 15% of strains. The heatmaps in Figure 1b demonstrated the gradual increase of sparsity (high absence of gene features among strains) from core genome to cloud genome in the gene feature matrix. The high sparsity of the pangenome matrix is caused by the large portion of cloud genes shared among extreme low amount of strains (Gaba et al., 2020). Different scopes of pangenome (core, soft core, shell, and cloud) were further utilized to train diverse learning models and interpretability of models was further used to yield biological insights of gene importance contributing to the isolation source.

**Table 1:** Distribution of genes across strains by pangenome classes.

Gene category	Strains (%)	Number of genes	Sparsity (%)
Core genes	99 ≤ strains ≤ 100	1,161	0.31%
Soft core genes	95 ≤ strains < 99	1,003	2.80%
Shell genes	15 ≤ strains < 95	3,229	39.76%
Cloud genes	0 ≤ strains < 15	96,029	99.43%
Total genes	0 ≤ strains ≤ 100	101,422	95.44%

### Importance of isolation source labels as informative signals

**Figure 2:**
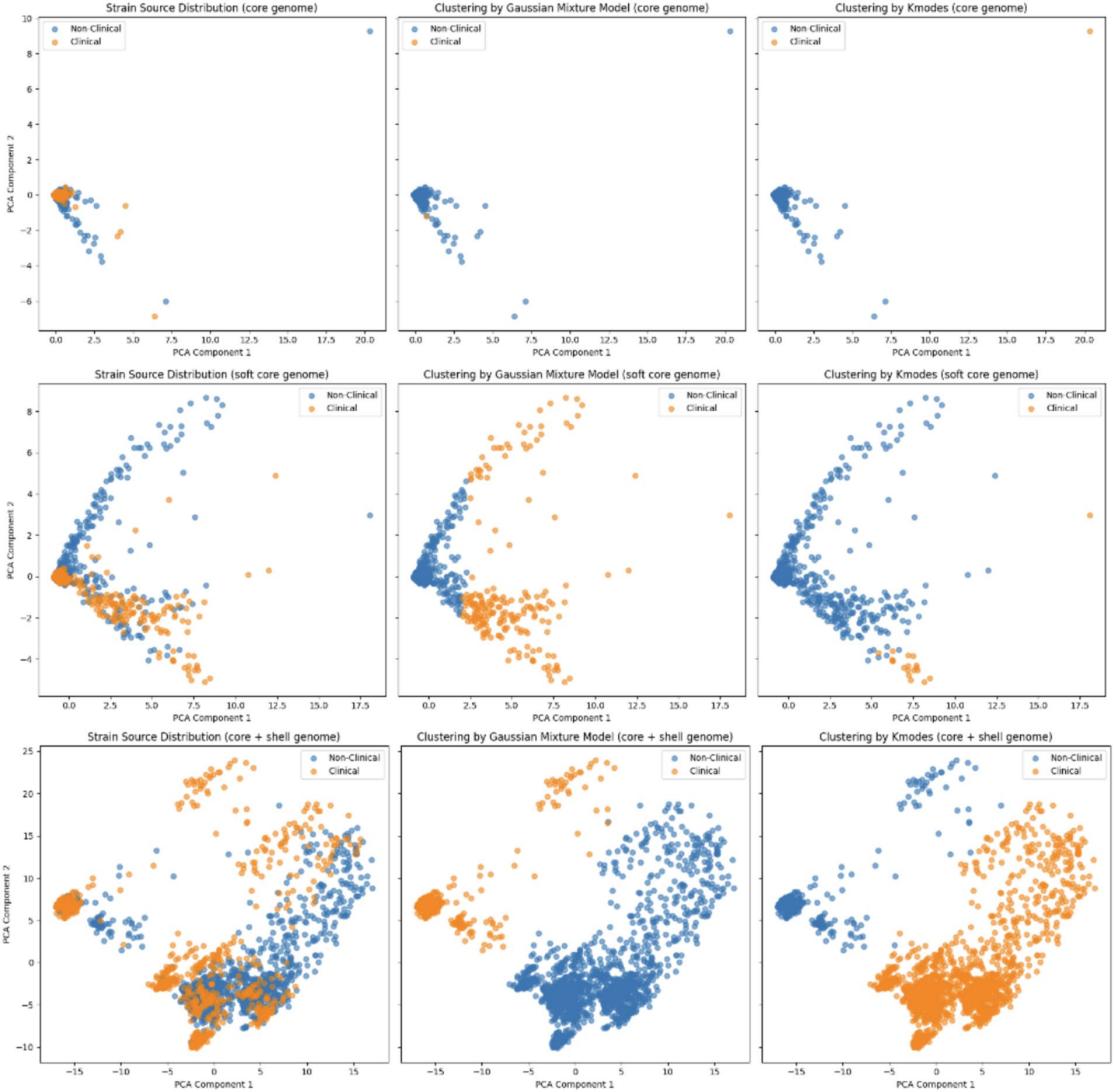
The PCA plots show the clustering of strains based on their source (clinical vs. environmental) using PCA components across three genomic categories: core genome (top row), soft core genome (middle row), and combined core + shell genome (bottom row). The left column represents the actual source distribution of strains, while the middle and right columns show clustering results using Gaussian Mixture Models and K-modes clustering, respectively. Orange dots represent clinical strains, and blue dots represent non-clinical strains. The differences in clustering patterns reveal insights into how genomic features influence the differentiation of clinical and non-clinical strains.

The left column of the Figure 2 represents the actual source distribution of *Vp* strains, which was visualized using PCA and categorized as clinical (orange dots) and environmental (blue dots), across three different pangenome matrices: core genome (top row), soft core genome (middle row), and core + shell genome (bottom row). These plots serve as the baseline to compare the clustering results from unsupervised clustering algorithms. Notably, as the number of gene features increased from the core genome through the soft core genome and finally to the core + shell genome, the separation between clinical and environmental strains became more distinctive based on PCA visualization (Figure 2). In the core genome, the two classes showed considerable overlap, which indicated the limited separability offered by 1,161 core pangenome genes. This was due to the fact that genes in the core genome were shared among 99% of *Vp*, and therefore *Vp* strains with core pangenome lacked the variability needed to effectively distinguish different clusters. As additional gene features were included in soft core and core+shell genome, two classes of strains were separated although they remained partially nested, which indicated the intricate relationships between gene features and isolation source.

Unsupervised clustering algorithms (GMM and K-modes) on three different pangenome matrices (core, soft core, core + shell) were then examined. Table 2 highlights the evaluation of unsupervised clustering algorithms (GMM and K-modes) across different pangenome matrix, and the results demonstrate the unsuitability of unsupervised clustering for separating isolation sources. Unsupervised clustering performed poorly regardless of the pangenome matrixes with consistently low recall and F1-scores, indicating limited effectiveness in distinguishing isolation source. The whole pangenome matrix was excluded from the unsupervised clustering because the algorithms failed to converge due to its high dimensionality and sparsity, both of which posed significant challenges to the optimization processes of unsupervised models. Similar poor performance of unsupervised clustering based on PCA-processed pangenome of Shiga-toxin producing *Escherichia.coli* was reported before (Im et al., 2021).

**Table 2:** Unsupervised clustering of clinical and nonclinical strains based on different genome classes.

Genome type	Model	Accuracy	Precision	Recall	F1-Score
Pangenome	GMM	N/Aᵃ	N/Aᵃ	N/Aᵃ	N/Aᵃ
	Kmodes	N/Aᵃ	N/Aᵃ	N/Aᵃ	N/Aᵃ
Core + Shell genome	GMM	0.574 ± 0.039	0.517 ± 0.113	0.387 ± 0.252	0.402 ± 0.170
	Kmodes	0.628 ± 0.000	0.714 ± 0.000	0.258 ± 0.000	0.379 ± 0.000
Soft core genome	GMM	0.566 ± 0.005	0.401 ± 0.270	0.078 ± 0.063	0.126 ± 0.101
	Kmodes	0.565 ± 0.007	0.733 ± 0.368	0.015 ± 0.008	0.030 ± 0.016
Core genome	GMM	0.560 ± 0.001	0.371 ± 0.435	0.001 ± 0.002	0.003 ± 0.003
	Kmodes	0.559 ± 0.002	0.188 ± 0.249	0.001 ± 0.002	0.003 ± 0.004

ᵃN/A indicates that the model failed to converge during training.

### Isolation source labels guided accurate classification and enabled the discovery of clinical-associated genes

Given the failure of unsupervised clustering methods to classify clinical versus environmental strains without isolation source labels, supervised classification approaches were subsequently investigated (Table 3). The core genome provided limited signals and all of these trained learning algorithms were not capable of distinguishing isolation sources (high false negative rate with recall ranging from 0.000 to 0.638). Additionally, AUC scores ranged from 0.5000 to 0.682, indicating performance close to random guessing. These results highlight the limited discriminatory power of the core genome for classification tasks. The addition of the soft core genome expanded the feature set by including genes shared among at least of 95% strains. This increase of gene features in soft core pangenome led to substantial improvements in model performance. RF achieved the highest performance, with an accuracy of 0.816 ± 0.022 and an AUC score of 0.893 ± 0.018, showcasing its robustness to handle added soft core gene features following performance of GBT (recall, 0.797 ± 0.028; AUC score 0.877 ± 0.024). The SVM was unable to distinguish isolation sources with low recall (0.235 ± 0.032) and F1-score (0.368 ± 0.035), indicating a high false negative rate and a substantial proportion of misclassified true positive samples. NB still underperformed compared to other classifiers, with a recall and AUC score of 0.579 ± 0.024. The results indicate that while the addition of soft-core genomic features enhanced separability, models like SVM and NB still required additional informative signals from the broader pangenome.

**Table 3:** Supervised classification evaluation across different pangenome classes.

Genome type	Model	Accuracy	Precision	Recall	F1-Score	AUC score
Core genome	SVM	0.560 ± 0.049	0.000 ± 0.000	0.000 ± 0.000	0.000 ± 0.000	0.617 ± 0.031
	Random forest	0.610 ± 0.033	0.666 ± 0.030	0.638 ± 0.027	0.598 ± 0.032	0.682 ± 0.032
	K-nearest neighbors	0.530 ± 0.052	0.635 ± 0.044	0.566 ± 0.025	0.476 ± 0.043	0.592 ± 0.033
	Gradient boosting trees	0.560 ± 0.049	0.280 ± 0.024	0.500 ± 0.000	0.358 ± 0.020	0.500 ± 0.000
	Gaussian Naive Bayes	0.504 ± 0.040	0.609 ± 0.041	0.549 ± 0.016	0.450 ± 0.026	0.549 ± 0.016
Soft core genome	SVM	0.647 ± 0.034	0.868 ± 0.073	0.235 ± 0.032	0.368 ± 0.035	0.836 ± 0.027
	Random forest	0.816 ± 0.022	0.811 ± 0.023	0.813 ± 0.023	0.812 ± 0.023	0.893 ± 0.018
	K-nearest neighbors	0.630 ± 0.033	0.679 ± 0.024	0.653 ± 0.028	0.619 ± 0.035	0.749 ± 0.031
	Gradient boosting trees	0.799 ± 0.026	0.795 ± 0.027	0.797 ± 0.028	0.795 ± 0.027	0.877 ± 0.024
	Gaussian Naive Bayes	0.538 ± 0.038	0.639 ± 0.044	0.579 ± 0.024	0.500 ± 0.030	0.579 ± 0.024
Core + Shell genome	SVM	0.862 ± 0.024	0.925 ± 0.028	0.749 ± 0.038	0.827 ± 0.022	0.941 ± 0.017
	Random forest	0.911 ± 0.017	0.911 ± 0.016	0.908 ± 0.018	0.909 ± 0.017	0.961 ± 0.010
	K-nearest neighbors	0.880 ± 0.013	0.877 ± 0.013	0.881 ± 0.014	0.878 ± 0.013	0.939 ± 0.012
	Gradient boosting trees	0.914 ± 0.014	0.913 ± 0.015	0.911 ± 0.015	0.911 ± 0.015	0.968 ± 0.011
	Gaussian Naive Bayes	0.580 ± 0.061	0.665 ± 0.056	0.616 ± 0.053	0.553 ± 0.069	0.616 ± 0.057
Whole pangenome	SVM	0.818 ± 0.021	**0.947 ± 0.026**	0.622 ± 0.039	0.749 ± 0.025	0.919 ± 0.019
	Random forest	0.916 ± 0.014	0.917 ± 0.014	0.911 ± 0.016	0.913 ± 0.014	0.961 ± 0.013
	K-nearest neighbors	0.852 ± 0.013	0.848 ± 0.014	0.853 ± 0.014	0.849 ± 0.013	0.920 ± 0.015
	Gradient boosting trees	0.914 ± 0.013	0.913 ± 0.014	0.911 ± 0.015	0.912 ± 0.014	0.969 ± 0.008
	Gaussian Naive Bayes	0.863 ± 0.031	0.874 ± 0.029	0.851 ± 0.029	0.857 ± 0.031	0.851 ± 0.029
Selected pangenome	SVM	0.907 ± 0.014	0.922 ± 0.017	0.861 ± 0.034	0.890 ± 0.018	0.962 ± 0.013
	Random forest	**0.919 ± 0.014**	0.920 ± 0.014	**0.916 ± 0.015**	**0.917 ± 0.014**	0.969 ± 0.010
	K-nearest neighbors	0.898 ± 0.018	0.897 ± 0.018	0.895 ± 0.019	0.895 ± 0.018	0.946 ± 0.010
	Gradient boosting trees	0.917 ± 0.015	0.916 ± 0.016	0.914 ± 0.016	0.915 ± 0.015	**0.971 ± 0.009**
	Gaussian Naive Bayes	0.913 ± 0.017	0.911 ± 0.018	0.911 ± 0.017	0.911 ± 0.018	0.929 ± 0.022

Bold values indicate the highest performance achieved for each specific metric within its respective pangenome category.

Shell pangenome sharing at least 15% *Vp* genomes features were further incorporated into the input data. The core + shell combination showed a clear improvement in classification performance across all models. RF and GBT both achieved remarkable results, with accuracy and AUC scores reaching 0.961 ± 0.010 and 0.968 ± 0.011, respectively, and recall 0.908 ± 0.018 and 0.911 ± 0.015, respectively. SVM showed substantial improvement, with an increased recall from 0.235 ± 0.032 to 0.749 ± 0.038 and increased AUC from 0.836 ± 0.027 to 0.941 ± 0.017, demonstrating that SVM benefitted significantly from the additional features. NB lag behind other models, with an AUC of 0.616 ± 0.057 and recall 0.616 ± 0.053. To further examine the value of accessory cloud genes (shared among less than 15% *Vp* genomes), whole pangenome was used as the input of learning models. Noticeably, SVM reported the highest precision 0.947 ± 0.026, indicating that the inclusion of accessory cloud genes led to a low false positive rate and benefit confident separation of isolation sources. Using the whole pangenome led to the highest performance of NB (all evaluation metrics exceeding 0.85). The prior assumptions of NB suggested that accessory cloud genes provide additional independent signals facilitating estimation of class probabilities. Both improved results on SVM and NB demonstrates that low-frequency cloud genes contain useful signals contributing the source differentiation despite the high sparsity.

**Figure 3:**
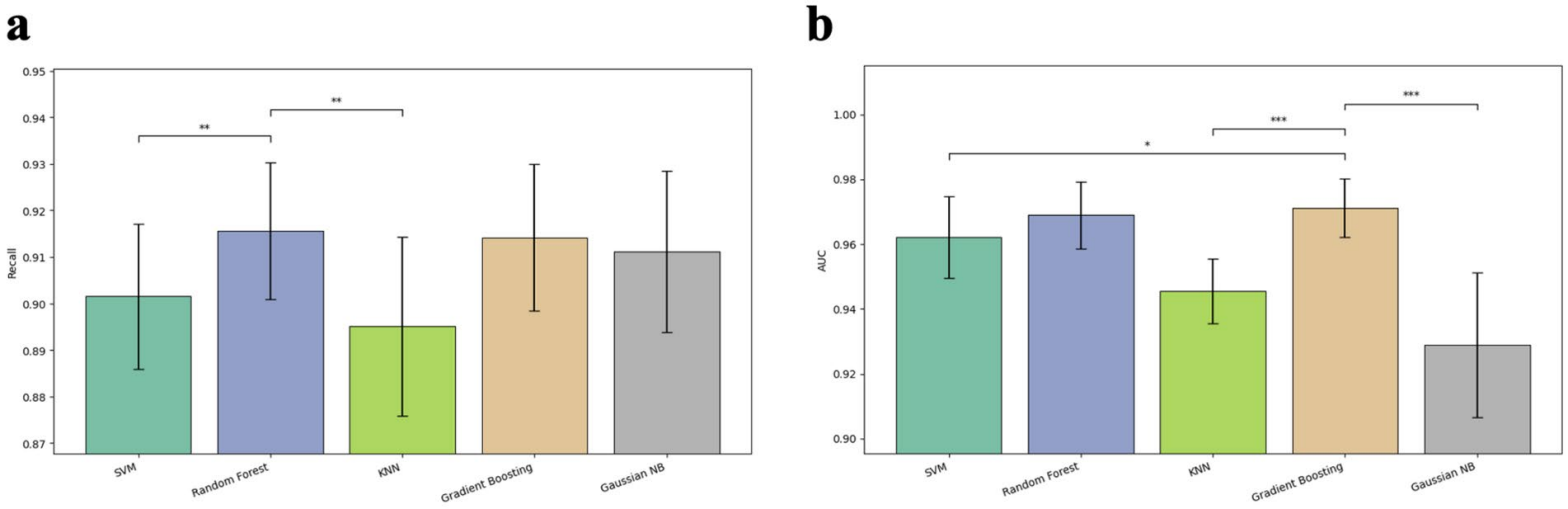
Model performance using selected pangenome. **(a)** Comparison of the models’ ability to correctly identify positive samples. RF demonstrate higher mean recall compared to SVM and kNN. **(b)** Comparison of the models’ discriminatory power. Gradient boosting and random forest achieve the highest AUC scores. Statistical significance between selected comparisons is indicated by asterisks (*p < 0.05, **p < 0.01, ***p < 0.001).

One of the major drawbacks of using high dimensional whole pangenome was substantial computational cost, particularly the training time and memory consumption (Table 4). Feature selection is crucial for enhancing both the performance and training efficiency of machine learning models, particularly for supervised classifiers (Li et al., 2017). The top 1936 gene features with importance were retrieved from RF feature importance analysis used to construct the selected pangenome. NB, which initially struggled with limited features, showed significant boosts in accuracy and AUC scores as more genomic information was included, reaching its best performance with the selected pangenome (precision: 0.911 ± 0.018, AUC: 0.929 ± 0.022). With selected pangenome as input data, false negative rate indicated by recall was significantly lower in RF, GBT and NB (0.916 ± 0.015, 0.914 ± 0.016, and 0.911 ± 0.017) than in SVM and KNN. Highest AUC score (0.969 ± 0.010 and 0.971 ± 0.009, respectively) of tree-based models (RF and GBT) were significantly higher compared with the rest of models (Figure 3). Additionally, model performance improvements, distinct training time decrease was also observed (RF 62.92 s to 3.35 s; GB 1034.74 s to 16.78 s) (Table 4), which indicated the power of selected features in model training efficiency.

**Table 4:** Computational cost of supervised classification across different pangenome classes.

Genome type	Model	Total time (s)	Peak memory (MB)
Core genome	SVM	55.28	30.44
	Random forest	8.98	8.16
	K-nearest neighbors	0.30	20.38
	Gradient boosting trees	4.34	29.71
	Gaussian Naive Bayes	0.08	18.10
Soft core genome	SVM	80.27	54.69
	Random forest	8.20	14.94
	K-nearest neighbors	0.33	35.48
	Gradient boosting trees	24.27	128.83
	Gaussian Naive Bayes	0.16	33.63
Core + Shell genome	SVM	238.48	131.18
	Random forest	8.58	36.93
	K-Nearest Neighbors	0.58	84.26
	Gradient boosting trees	70.16	716.06
	Gaussian Naive Bayes	0.45	83.61
Whole pangenome	SVM	3131.74	2595.34
	Random forest	73.68	691.18
	K-nearest neighbors	7.70	1534.89
	Gradient boosting trees	1167.78	12962.10
	Gaussian Naive Bayes	7.56	1570.86
Selected pangenome	SVM	40.90	39.08
	Random forest	4.98	13.38
	K-nearest neighbors	0.28	32.04
	Gradient boosting trees	25.94	248.04
	Gaussian Naive Bayes	0.16	30.10

### Feature importance ranking uncovered genes contributing to the classification of isolation sources

**Figure 4:**
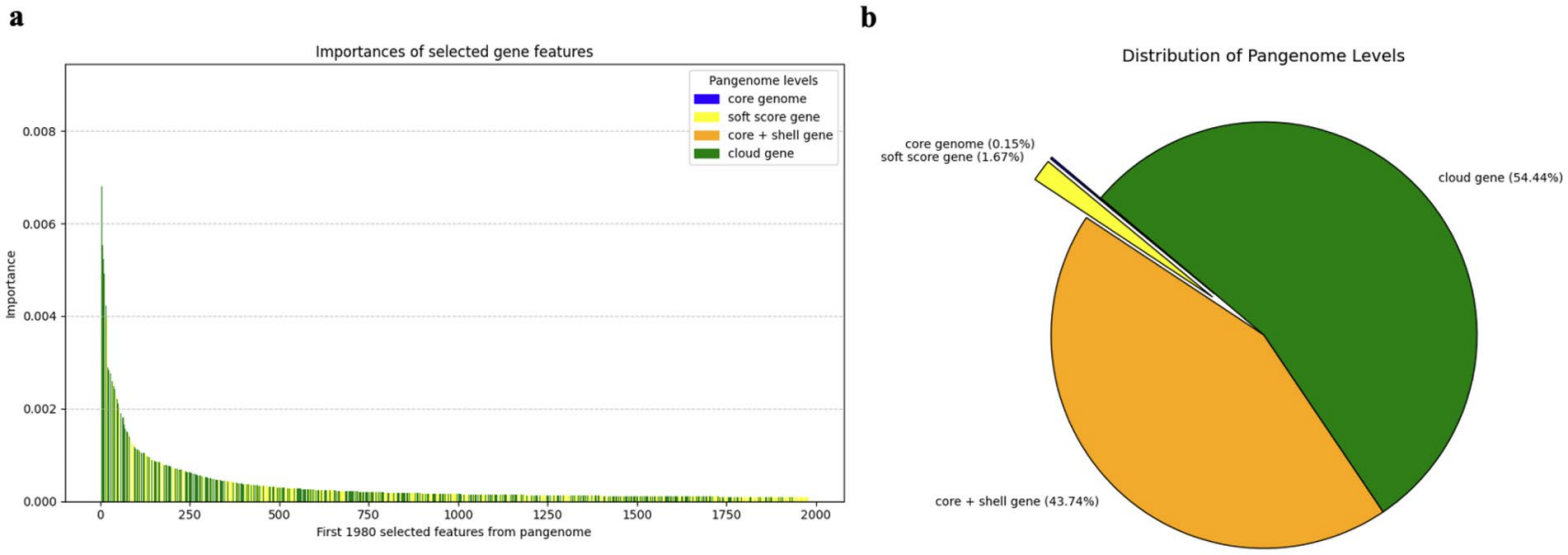
Overview of selected gene features from pangenome. **(a)** Importance of selected gene features: the importance weights of the top 1980 gene features as identified by a random forest model. **(b)** Distribution of pangenome levels is illustrated in a pie chart.

To gain biological insights into the classification of clinical versus environmental strains, it is essential to employ interpretable models that can highlight the genomic features driving the classification and predictions. Interpretability enables the identification of key genetic signals and provides a deeper understanding of the underlying biological mechanisms. To achieve this, RF feature importance analysis was conducted. The analysis of selected gene features from the pangenome highlighted the critical role of non-core genes, particularly cloud genes, in differentiating clinical versus environmental strains. Among the top-ranking gene features contributing to isolation source differentiation, multiple annotated virulence-associated genes were highlighted, including but not limited to *sct*C5 (type III secretion system secretin, rank 3 in annotated genes), *vir*F (virulence regulon transcriptional activator, rank 6 in annotated genes), and *tdh*1 (thermostable direct hemolysin 1 rank 11 in annotated genes), underscoring their biological relevance to pathogenic potential. Besides, genes identified as key contributors to *Vp* isolation source difference included but not limited to *yopJ*, *flgH1*, and *hlyC2*. Figure 4a shows the feature importance rankings generated by the RF model, where the top 1,980 gene features were identified based on their contribution to the classification efficiency. A significant proportion of these top-ranking features were classified as cloud genes, indicating their pivotal role in distinguishing strain types. This finding is supported by the distribution of pangenome levels in Figure 4b, where cloud genes constitute the majority (54.44%), followed by shell genes (43.74%) soft core genes (1.67%) and core genes (0.15%).

These results underscore the limited contribution of core genes and emphasize the essential role of cloud genes in capturing strain-specific gene variation because cloud genes are typically considered the most dynamic component of bacterial pangenomes, frequently associated with horizontal gene transfer, mobile genetic elements, and recent evolutionary events (Bazin et al., 2020; Gautreau et al., 2020). The RF model’s feature importance weights align closely with the genomic variability inherent in cloud genes, highlighting their significant impact on classification. Together, these findings demonstrate that the non-core pangenome, particularly cloud genes, is indispensable for effectively distinguishing clinical from environmental strains, supporting the need to consider the entire pangenome in classification models.

### Convolutional neural networks further highlighted regions in accessory cloud genes in pangenome

As illustrated by RF feature importance analysis, the cloud genes within the pangenome play a significant role in pathogenic potential prediction. Unlike core genes, which are conserved across strains, cloud genes are highly variable and are often associated with functions such as adaptation, pathogenicity, and resistance (Sonnenberg et al., 2020a). These characteristics make it essential to use each gene feature in the entire pangenome to fully capture the genetic variation that contributes to pathogenic potential. Incorporating the entire pangenome ensures that potentially crucial information, particularly from cloud genes, is not skipped by classic machine learning models. MLP and CNN models were then intentionally implemented to utilize the full set of gene features, which enabled the process to assess whether predictive signals could be extracted from the high-dimensional pangenome without feature selection like prior well-performing RF.

**Figure 5:**
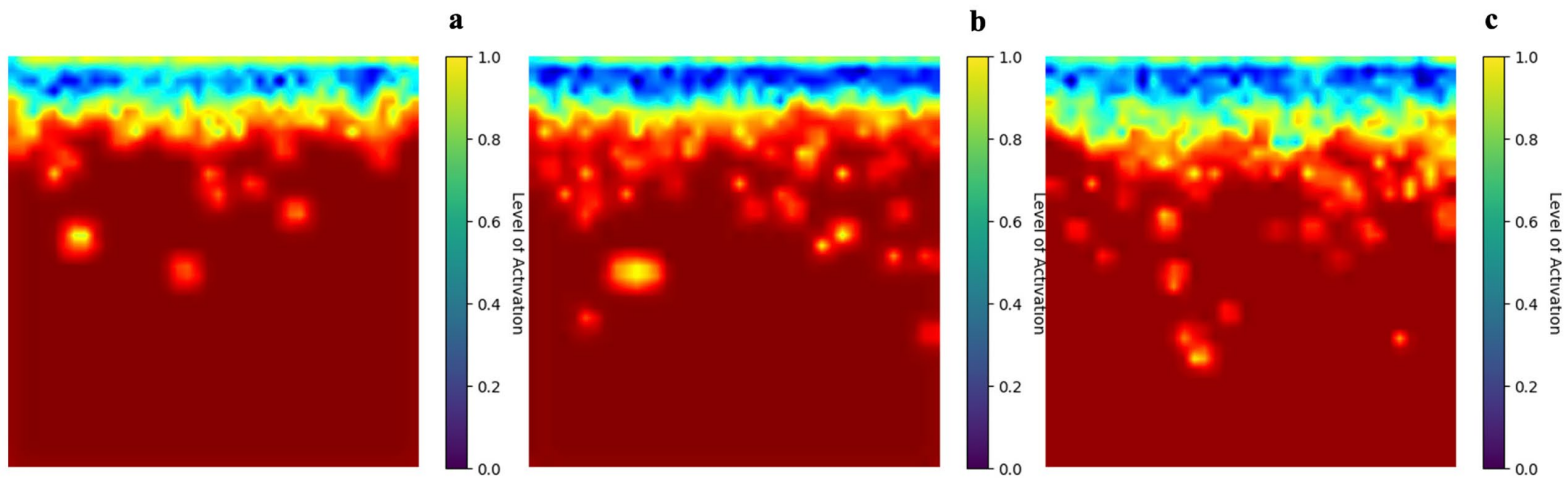
Exemplary Gradient-weighted Class Activation Mapping (Grad-CAM) heatmaps highlighting genomic regions within the clinical Vp whole pangenome that received high model attention for three representative genomes: **(a)** GCF_000430425.1 (ASM43042v1), **(b)** GCF_000489695.1 (strain S138), and **(c)** GCF_000489875.1 (strain S129).

MLP was initially employed to predict isolation source using whole pangenome as the baseline. However, MLP struggled with the sparsity inherent in the pangenome matrix (Table 5). This sparsity, characterized by the disproportionate distribution of non-zero entries across the matrix, diluted the contribution of cloud gene signals, resulting in limited performance. The inability of MLP to effectively isolate and amplify the sparse signals from the cloud genes highlighted the need for a more sophisticated approach. To address the high sparsity nature of cloud genes, the use of CNN was motivated not by spatial structure in genomic data, but by its ability to efficiently capture localized patterns and improve learning from high-dimensional sparse feature spaces. This ability makes CNNs particularly suited for pangenome signal processing, where critical signals are scattered across the matrix in a sparse distribution. As demonstrated in Table 5, CNNs outperformed MLP in classifying clinical and environmental strains, achieving a recall and AUC score of 0.911 ± 0.018 and 0.968 ± 0.008, respectively. These results highlight the robustness of CNNs in handling complex, sparse datasets while preserving and amplifying meaningful signals. By enabling the comprehensive analysis of the pangenome, CNNs not only achieved superior classification performance but also underscored the importance of incorporating the entire genetic spectrum, including the highly variable cloud genes, in predictive models. This study illustrates the potential of CNNs as a powerful tool working with high-dimensional and sparse datasets like the pangenome matrix. The Grad-CAM analysis of the CNN further corroborated the findings from the RF feature importance analysis, highlighting the critical role of non-core genes in classifying clinical versus environmental strains. As depicted in Figure 5, the heatmaps show the regions of the pangenome matrix where the model’s attention was most focused during classification. The areas of highest activation aligned with the non-core pangenome regions, particularly the cloud genes. This focus on non-core genomic features reinforced the importance of these highly variable genes in capturing strain-specific characteristics associated with pathogenic potential, which are often absent in the more conserved core genome. The alignment between the Grad-CAM results and the RF feature importance weights emphasizes the consistent and indispensable contribution of non-core genes to assess pathogenic potential, validating their biological relevance and the necessity of considering the entire pangenome in predictive models.

**Table 5:** Deep learning classification evaluation on whole pangenome.

Model	Accuracy	Precision	Recall	F1-Score	AUC score
CNN	0.911 ± 0.018	0.912 ± 0.018	0.911 ± 0.018	0.910 ± 0.018	0.968 ± 0.008
MLP	0.871 ± 0.033	0.872 ± 0.032	0.871 ± 0.033	0.870 ± 0.034	0.930 ± 0.023

## Discussion

Given its prevalence and negative impacts on both public health and the worldwide seafood industry, rapid and effective methods for characterizing *Vp* are needed to detect and identify pathogenic *Vp* strains. Identifying features that can be used to timely separate clinical strains from environmental strains help better understand the evolution of *Vp* strains and their behavior in human body and seafood production environment. Although established virulence markers such as *tdh* and *trh* are widely used in pathogenic *Vp* serotyping and detection, previous studies have evidenced that these markers alone are not sufficient for differentiating clinical from environmental strains (Raghunath, 2015; Ronholm et al., 2016). This study addressed the imperative needs for rapid and accurate tools to differentiate clinical from environmental *Vp* strains by integrating pangenome information with advanced machine learning methods. Throughout the evolutionary path of *Vp*, various genes associated with environmental adaptation have emerged, some of which might be associated with direct or indirect virulence-related properties (Beceiro et al., 2013). The latest comprehensive pangenomic analysis of *Vp* ST3 strains demonstrated how genetic divergence, selective pressures, and accessory gene acquisition during evolution facilitated its adaptation to the distinct local marine climate while also contributing to its virulence and ecological fitness (Campbell et al., 2024). In another study, Jana et al. (2022) emphasized that newly evolved (acquired through horizontal gene transfer and localized within auxiliary modules or near DNA mobility elements) virulence-associated genes in the *Vp* pangenome are rare but essential in contributing its pathogenicity. To capture sparse but useful signals from the pangenomic matrix during the evolutionary trace of *Vp*, different layers of pangenome were developed and fit into learning models. Results from this study demonstrated that incorporating more genes from the cloud gene component of the pangenome led to improved model performance (Table 3), which underscored the significance of newly evolved genes within the *Vp* pangenome, particularly those belonging to accessory pangenomic regions.

In the context of biological interpretability, RF feature importance analysis provided deterministically ranked critical genes contributing the pathogenic potential of *Vp* whereas CNN Grad-CAM analyses highlighted the regions of the pangenome potentially associated with *Vp* virulence. Both approaches consistently identified non-core genes, particularly cloud genes, as the key drivers of classification. These genes are likely associated with strain-specific virulence and adaptability traits, making them prime targets for further functional and experimental validation. Given to the higher resolution of single gene level contribution to the virulence determination in RF compared with CNN (lost graduality due to gradient upscaling), the top 1936 genes with highest weight from RF was analyzed. Among these, *vspR*, a transcriptional regulator, emerged as a significant contributor. According to Davies et al. (2012), *vsp*R is a transcriptional inhibitor that represses the expression of the major pandemic island in *Vibrio cholerae* and the disruption of this gene recapitulate the intestinal colonization defect in TarB-mutant *V. cholerae*, which was supposed to fail in mouse intestine colonization. In this study, *vspR* was ranked as the top one gene feature with the highest weight contributing the pathogencity determination of *Vp*. Consistent with substantial previous evidence, *tdh1* was found to play a significant role in determining the virulence of *Vp* further emphasizing the advantages of utilizing *tdh+* strains in research (Liao et al., 2017; Liu et al., 2023; Sami et al., 2022; Wang et al., 2018). Additionally, several genes associated with the Type 3 Secretion System (T3SS) including sctC5 type III secretin and type III effector protein encoded gene *yop*J were also highly ranked, suggesting the critical involvement of type III secretion system in the pathogenicity and host interaction mechanisms of *Vp*. Another top-ranked gene, *flgH1*, which encodes a structural component of the flagellar apparatus, underscored the importance of virulence factors associated with motility in colonization and persistence. These findings summarized in Table 6, highlight the pivotal role of specific genes in the pathogenicity of *Vp* and provide valuable insights for future research direction. Alongside these findings, *hlyC* 2 encoding Hemolysin-activating lysine-acyltransferase, an accessory gene from cloud genome, was also identified among the ranked critical features, which suggested that low-frequency, accessory genes in *Vp* cloud genome may contribute to strain-specific differences in isolation source.

**Table 6:** Ranking of the annotated genes based on their contribution to classification of clinical and environmental strains (virulence-associated genes were highlighted in red).

Gene name	Product	CP	NCP	Pangenome level	Order
vspR	Transcriptional regulator VspR	79.10%	16.77%	Shell	1
intA	Prophage integrase IntA	36.51%	5.50%	Shell	2
sctC5	Type 3 secretion system secretin	52.93%	9.02%	Shell	3
csy3	CRISPR-associated protein Csy3	51.89%	8.03%	Shell	4
pcaF	Beta-ketoadipyl-CoA thiolase	28.70%	8.75%	Shell	5
virF	Virulence regulon transcriptional activator VirF	36.05%	3.43%	Shell	6
pflD	Trans-4-hydroxy-L-proline dehydratase	46.27%	18.67%	Shell	7
ssaV	Secretion system apparatus protein SsaV	35.36%	4.15%	Shell	8
hns 2	DNA-binding protein H-NS	36.17%	12.80%	Shell	9
fdtA	TDP-4-oxo-6-deoxy-alpha-D-glucose-3,4-oxoisomerase	46.38%	15.96%	Shell	10
tdh1	Thermostable direct hemolysin 1	29.62%	2.71%	Core	11
nikE	Nickel import system ATP-binding protein NikE	36.17%	13.35%	Shell	12
rbsC	Ribose import permease protein RbsC	28.59%	61.95%	Shell	13
ptsG 1	PTS system glucose-specific EIICB component	45.35%	67.72%	Shell	14
atpB 2	ATP synthase subunit a	52.58%	13.35%	Shell	15
kce 2	3-keto-5-aminohexanoate cleavage enzyme	31.34%	8.48%	Shell	16
gmd	GDP-mannose 4,6-dehydratase	46.50%	15.33%	Shell	17
yscU 2	Yop proteins translocation protein U	36.17%	13.62%	Shell	18
ddpC	Putative D, D-dipeptide transport system permease protein	36.17%	13.53%	Shell	19
nadB	L-aspartate oxidase	70.49%	47.07%	Shell	20
purH	Bifunctional purine biosynthesis protein PurH	71.64%	90.26%	Shell	21
hlyC 2	Hemolysin-activating lysine-acyltransferase HlyC	10.91%	7.30%	Cloud	22
hslJ	Heat shock protein HslJ	85.30%	63.12%	Shell	23
rep 2	ATP-dependent DNA helicase Rep	13.43%	1.71%	Cloud	24
pctC	Methyl-accepting chemotaxis protein PctC	80.60%	92.70%	Shell	25
hlyB	Alpha-hemolysin translocation ATP-binding protein HlyB	9.74%	7.48%	Cloud	26
dctD 3	C4-dicarboxylate transport transcriptional regulatory protein DctD	69.69%	39.13%	Shell	27
coaBC	Coenzyme A biosynthesis bifunctional protein CoaBC	29.39%	69.52%	Shell	28
glpE 2	Thiosulfate sulfurtransferase GlpE	85.65%	58.43%	Shell	29
brnQ 2	Branched-chain amino acid transport system 2 carrier protein	81.17%	54.91%	Shell	30
intS	Prophage integrase IntS	25.75%	6.67%	Shell	31
fcl	GDP-L-fucose synthase	46.73%	15.51%	Shell	32
pctC 1	Methyl-accepting chemotaxis protein PctC	4.13%	0.36%	Cloud	33
bcsZ	Endoglucanase	14.70%	40.85%	Shell	34
cysG 4	Siroheme synthase	97.49%	96.03%	Shell	35
rhtC 3	Threonine efflux protein	25.37%	8.03%	Shell	36
ureA	Urease subunit gamma	36.05%	13.53%	Shell	37
coaD	Phosphopantetheine adenylyltransferase	85.76%	60.87%	Shell	38
yiaV 2	Inner membrane protein YiaV	91.27%	71.69%	Shell	39
yopJ	Effector protein YopJ	51.32%	8.57%	Shell	40
lamB	Maltoporin	31.00%	67.90%	Shell	41
flgH 1	Flagellar L-ring protein	80.25%	91.97%	Shell	42

CP, Clinical strain presence rate; NCP, Non-clinical presence rate.

**Figure 6:**
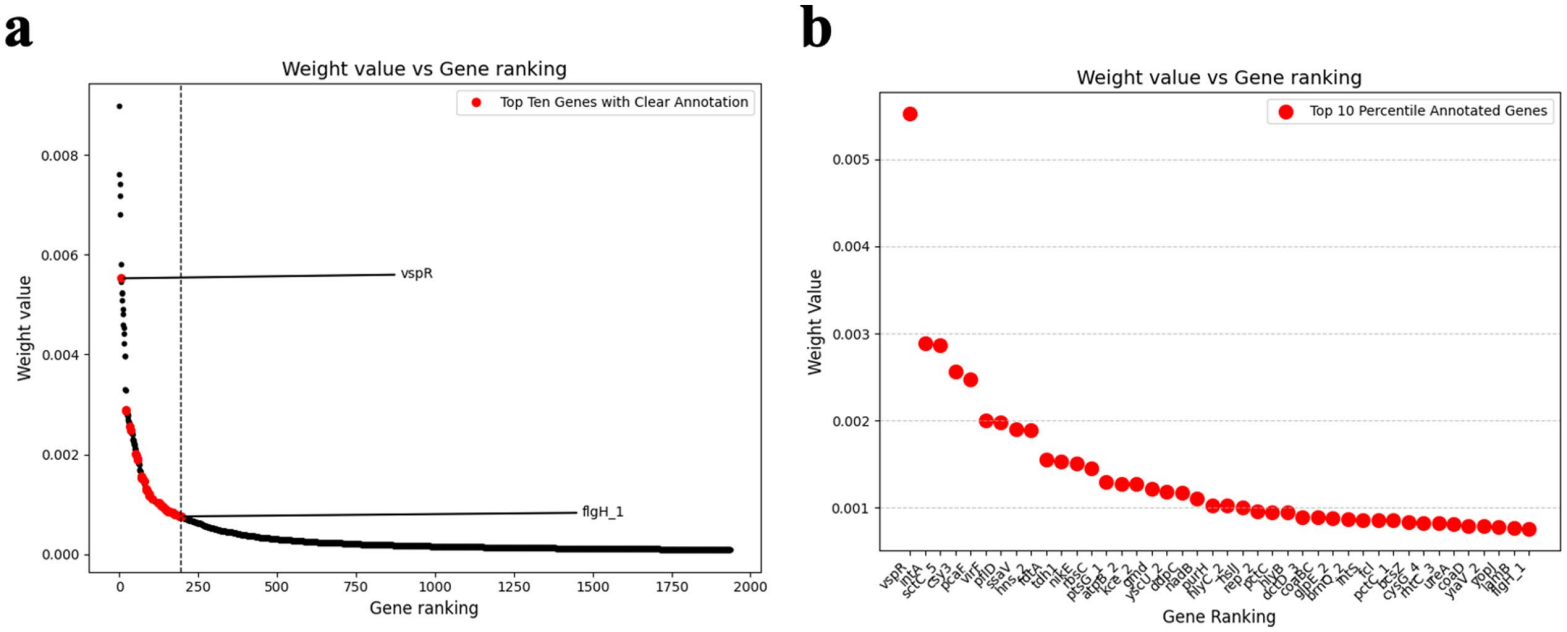
**(a)** Gene feature weight values assigned to genes by the Random Forest model across different rankings. The *x*-axis represents the gene ranking (ordered by weight value), and the *y*-axis shows the corresponding weight values. The red points highlight the genes in the top 10 percentile with clear annotations from *prokka*. *vspR* and *flgH_1* were annotated on the graph for reference the start and end annotated gene list, respectively. **(b)** The zoomed-in view of the top 10 percentile of annotated genes from the ranking. The *x*-axis lists the gene names, and the *y*-axis displays their respective weight values.

It was noteworthy to point out that a significant proportion of virulence-associated genes in *Vp* remained functionally unknown (failed being annotated by Prokka) and these genes usually do not serve as targets or usable references in conventional gene serotyping due to lack of sufficient information. Among the top 1936 genes contributing to isolation source difference as the proxy to pathogenic potential, 1,528 were categorized as functionally unknown (78.93%) (Figure 6), emphasizing a large gap in gene annotation of their roles in pathogenicity. Notably, in the top 200 genes, 157 were also identified as functionally unknown (79.00%), further underscoring the need for focused research on these unexplored genes to uncover novel mechanisms driving virulence. These facts underscored the advantages of ML/DL models lies in their ability to leverage these functionally unknown genes to accurately predict the pathogenic potential of *Vp*, which are capable of uncovering hidden patterns and relationships that traditional methods might overlook. Single-nucleotide polymorphism (SNP) and k-mer based machine learning approaches have already been successfully applied to predict diverse types virulence in pathogenic bacteria (Antimicrobial resistance, virulence factor expression and general virulence prediction) (Allen et al., 2021). Compared with these sequence-based methods, pangenome-based machine learning methods capture broader gene presence–absence patterns, accessory gene variation, and structural signals to bridge the gap due to unannotated genes. The findings of this study provided critical insights into the application of machine learning approaches for both diagnostics and virulence discovery of *Vp* using whole-genome sequencing (WGS) data.

Although this study has demonstrated promising performance to identify isolation sources and virulence potential of *Vp* using pangenome, several aspects warrant further careful interpretation. For instance, pangenome matrix only records whether coding sequences (CDSs) are present or absent, and therefore cannot capture sequence-level mutations (such as SNPs and indels) that may contribute to differences between isolation sources. Additionally, a key limitation of the Grad-CAM analysis is that the ordering of features is artificial and does not reflect their true positions in the genome. As a result, any local patterns identified by the model should be interpreted as computational groupings rather than biologically meaningful genomic structures. Moreover, we acknowledge that environmental isolates may also retain pathogenicity, and isolation source is not the key sole factor to determine pathogenicity. In the meantime, it has been known that genetic adaptation to specific environments is linked to bacteria pathogenicity (Castelli et al., 2023; Im et al., 2021). Nemati et al. (2024) indicated that clinical origin was a statistically robust proxy for human health risk when modeling pathogenic potential. Therefore, isolation sources were used as the proxy of pathogenic potential instead of deterministic pathogenicity labels in this study. Nevertheless, by integrating pangenome-based features with advanced computational methods, this study sheds light for future rapid and accurate tools to assess pathogenic potential of pathogenic bacteria. In modern pathogenic surveillance systems, minimizing computational time intake is critical for timely detection and response (Gardy and Loman, 2018). For real-time production-grade deployment, classical machine learning models offer a more cost-effective solution than deep learning approaches, as exemplified by RF and NB achieving substantially faster training time and lower memory requirement (e.g., 4.98–73.68 s runtime and 13.38–691.18 MB memory), whereas DL models typically require GPU acceleration and therefore lead to substantially higher training time and memory overhead in hours depending on model architectures. Future direction includes but not limited to building and validation ML-driven surveillance systems by developing lightweight, scalable models for real-time pathogen detection and risk assessment.

## Conclusion

Unsupervised learning models were unable to effectively distinguish between clinical and environmental *Vp* strains, whereas supervised machine learning approaches enabled accurate source differentiation. Model performance consistently improved as gene features expanded from the core genome to the full pangenome, highlighting the importance of accessory genomic content in capturing pathogenic signals. Several supervised machine learning and deep learning models achieved strong discriminative performance, with area under AUC scores higher than 0.95 using whole or selected pangenome feature sets. This demonstrates the promising application of ML and DL in food safety areas. Most impotently, RF and CNN Grad-CAM facilitated the identification of gene features contributing to strain isolation source difference. In addition to the well-known virulence-associated genes, such as *tdh1* the identification of genes like *VspR* provide new insight and potential new target for the development of molecular tools for rapid detection of *Vp* of clinical importance, which contributes to improved public health outcomes and food industry practices.

## Data Availability

The original contributions presented in the study are included in the article/Supplementary material, further inquiries can be directed to the corresponding author.

## Footnotes

1
https://www.ncbi.nlm.nih.gov/datasets/genome/

## Supplementary material

SUPPLEMENTARY Figure 1 Workflow for pangenome-based classification and biological insight discovery. Complete *Vp* genomes were retrieved from NCBI and processed through two parallel parts. Complete *Vp* genomes were annotated using Prokka and subsequently used for pangenome construction with Roary to generate gene presence–absence matrices. Isolation source information (environmental vs. clinical) was extracted and curated to define ground truth labels. These data were then integrated for machine learning model training and evaluation to classify strain origin. Finally, model outputs were analyzed to extract biologically relevant insights, including genes associated with pathogenic potential.
