# Supplementary material for: Interpretable learning algorithms enable pathogenic potential assessment and virulence-associated gene discovery of Vibrio parahaemolyticus
Source: Front Microbiol. 2026 May 20;17:1832130. doi: 10.3389/fmicb.2026.1832130 (PMC13234855; doi:10.3389/fmicb.2026.1832130)
Supplement: Supplementary file 1 [file Data_sheet_1.docx]

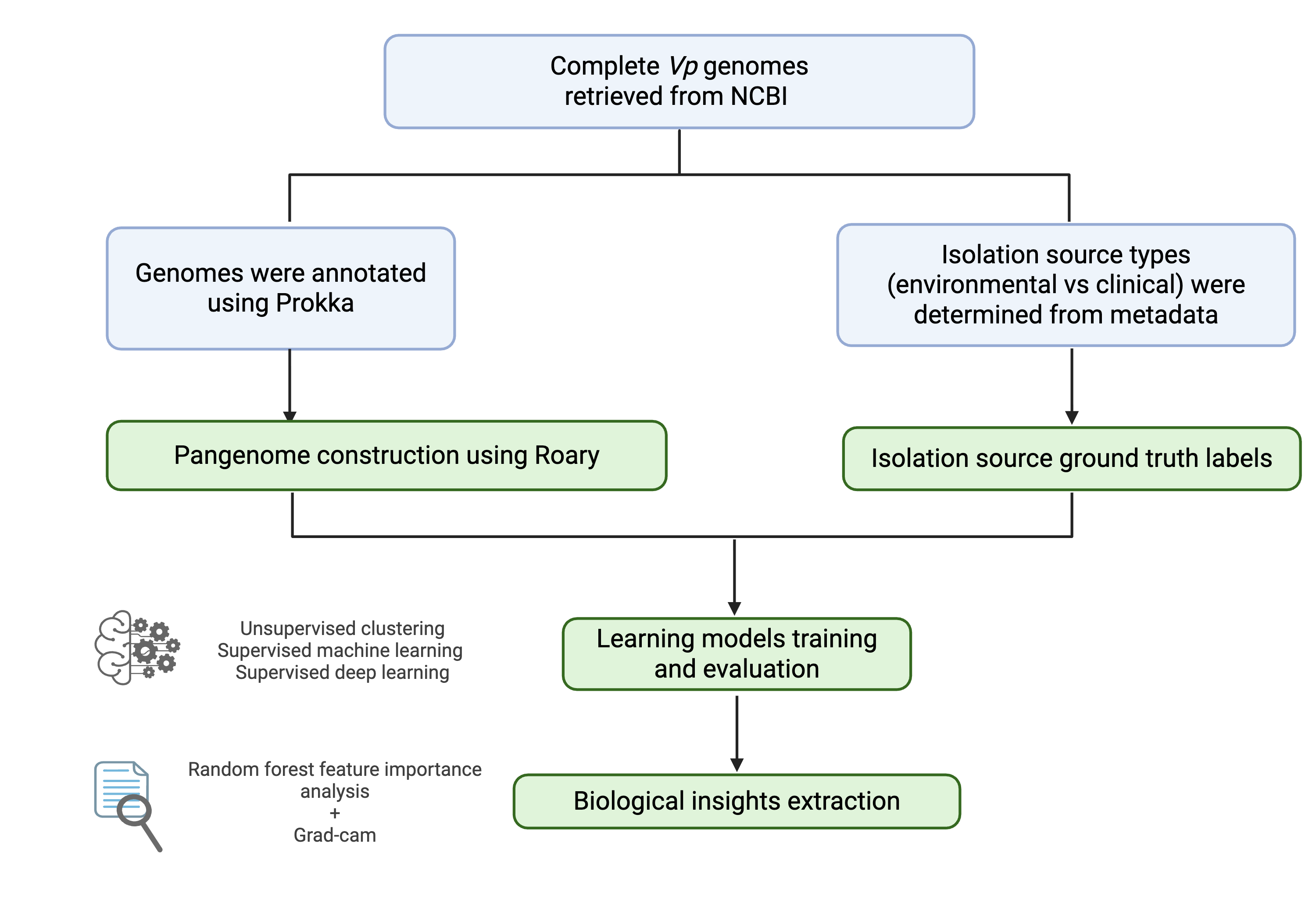


**Supplementary Figure 1.** Workflow for pangenome-based classification and biological insight discovery. Complete *Vp* genomes were retrieved from NCBI and processed through two parallel parts. Complete *Vp* genomes were annotated using Prokka and subsequently used for pangenome construction with Roary to generate gene presence–absence matrices. isolation source information (environmental vs. clinical) was extracted and curated to define ground truth labels. These data were then integrated for machine learning model training and evaluation to classify strain origin. Finally, model outputs were analyzed to extract biologically relevant insights, including genes associated with pathogenic potential.
